# Ecological succession of adult necrophilous insects on neonate *Sus scrofa domesticus* in central North Carolina

**DOI:** 10.1371/journal.pone.0195785

**Published:** 2018-04-12

**Authors:** Angela Cruise, David W. Watson, Coby Schal

**Affiliations:** 1 Department of Entomology and Plant Pathology, North Carolina State University, Raleigh, North Carolina, United States of America; 2 W.M. Keck Center for Behavioral Biology, North Carolina State University, Raleigh, North Carolina, United States of America; Natural Resources Canada, CANADA

## Abstract

The necrophilous insect fauna on carcasses varies seasonally and geographically. The ecological succession of insects arriving to decaying neonate pig carcasses in central North Carolina during late summer was sampled using a novel vented-chamber collection method. We collected six blow fly species, flesh flies, house flies and 10 beetle taxa, including four species of scarab beetles. Necrophilous fly activity dominated the early decomposition stages, whereas beetle numbers remained low until day 4. By day 7, more than 50% of the pig carcasses were skeletonized and they attracted few insects. Differences in the taxa and successional patterns documented in this experiment and a previous study in the same location highlight the ecological variation in such investigations, and underscore the need for standardization, as well as for ecological succession studies on finer geographic scales.

## Introduction

The documentation of ecological succession of the local fauna on decomposing model organisms is a critical component of forensic entomology research. Necrophilous insects are attracted to and colonize a decomposing body–whether human or carrion–in a predictable sequence [1–5]. During the pre-colonization phase, the insects detect, locate and evaluate the remains, followed by the colonization and post-colonization phase as the remains are consumed [6]. In the practice of forensic entomology, this predictable successional pattern, along with environmental parameters such as temperature, are used to calculate a postmortem interval (PMI), or perhaps more accurately, time since insect arrival. However, the taxa attracted, their development times, and the successional sequence itself vary by host, season, and geographic region; they may even vary on a microgeographic scale, such as between urban and rural environments within the same city [5, 7–10].

Seasonal and geographic variability in insect succession are frequently addressed in the forensic literature. While certain forensically significant insect taxa like *Musca domestica* are cosmopolitan, the vast majority of necrophilous insect taxa have more limited distributions. This natural variation in the distribution of taxa obviously influences the successional pattern across locations. Certain invasive necrophilous species, such as the blow fly *Chrysomya rufifacies*, may even influence the typical arrival pattern of native species [11]. It is important to document the succession of local fauna and reevaluate it in light of species invasions or climate-driven changes in species distributions. Seasonal differences must also be considered, as different species are indicative of different seasons, even in the same locality. For example, *Calliphora vicina* is a cool weather species in central North Carolina, most commonly sampled in the fall and winter and absent in the summer [12].

This research had three primary objectives. First, we aimed to document the succession of adult necrophilous insects in central North Carolina. Because ecological succession has been described on juvenile pig cadavers in this locality [12], our use of neonate pigs extends these findings to a smaller decomposition model of ecological succession. Second, in response to difficulties collecting large samples from small pigs, we developed a “vented-chamber” method to document succession. This passive sampling method has significantly increased the number of sampled insects compared to traditional sampling methods like the aerial sweep net [13, 14]. Moreover, because this trap eliminates all but olfactory cues, this study represents the first time, to our knowledge, that ecological succession is documented based solely on olfaction. Finally, in our preliminary fieldwork, we consistently found beetles in the family Scarabaeidae (AMC personal observations) on carrion, so this study aimed to document the arrival pattern of these dung-associated beetles. These beetles are rarely included in documentation of succession, as they are generally considered incidental fauna at a body, rather than primary colonizers and decomposers of forensic significance [15].

## Methods

### Ethics statement

Naturally stillborn pigs (*Sus scrofa domesticus*) were acquired from North Carolina State University’s Swine Educational Unit. The use of these neonate pigs in field studies of decomposition is exempt from approval from the Institutional Animal Care and Use Committee.

### Experimental animals

Stillborn pigs (*Sus scrofa domesticus*) weighed ~1.5 kg and were placed in a freezer immediately after birth and remained fully frozen until they were placed in the field. This prevented early decomposition and ensured that all pigs were the same temperature at the start of the experiment. Pigs at -12°C reached ambient temperature in ~4.5 hrs in full sunlight (AMC personal observations), so they were at ambient temperature when sampling commenced 24 hrs after placement in the field (below).

### Study site

This experiment was conducted during September 2015 in an open field at North Carolina State University’s Lake Wheeler Road Field Lab in Raleigh, NC (35.731424, -78.667759). Pigs were positioned at the northern edge of a closely mowed field, where they experienced full daytime sun.

**Fig 1:**
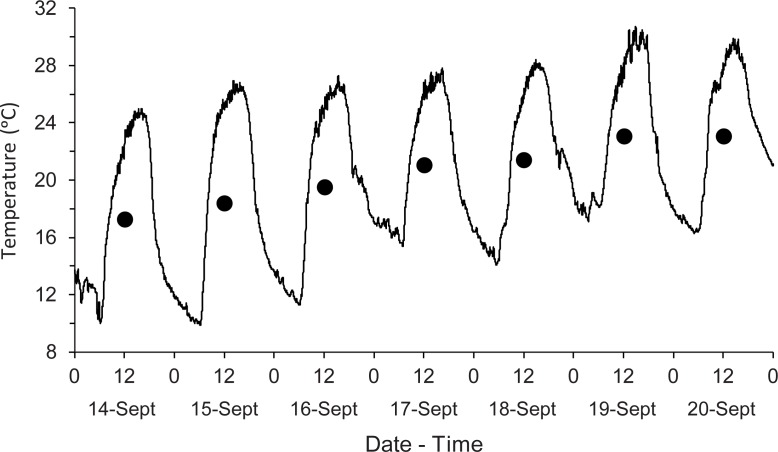
Minute and daily average ambient temperature (°C) near the field research site during the experiment. The symbols indicate the average daily temperature. Temperature data were acquired from the State Climate Office of North Carolina’s Lake Wheeler Road Field Lab weather station. There was no measurable precipitation during this period.

Weather conditions were ideal for a succession experiment, with no precipitation and a gradual increase in ambient temperature during the 7 sampling days (Fig 1). The average ambient temperature during the 7-day experiment was 20.65 ± 0.88°C (SEM). Winds were predominantly Northeasterly and Southeasterly throughout the experiment. We minimized between-pig variation by using pigs of the same size, body temperature, and level of concealment at the field edge.

### Field methods

During the afternoon of the first day of the experiment (defined as day 0), eight sites were established 25 m apart in the experimental field. At each site, soil was excavated and spread ~ 4 cm deep atop a standard plastic cafeteria tray (35 cm x 45 cm). Eight fully frozen pigs were placed on the 8 soil-covered trays. This arrangement allowed us to move each pig into the vented-chamber (described below) during sampling periods. Pigs were photographed each day prior to sampling to document insect activity and the stage of decomposition. Stages of decomposition were as defined by Kreitlow [16]. When not sampled, the carcasses were protected from scavengers within cages constructed of poultry netting that allowed for normal insect colonization.

**Fig 2:**
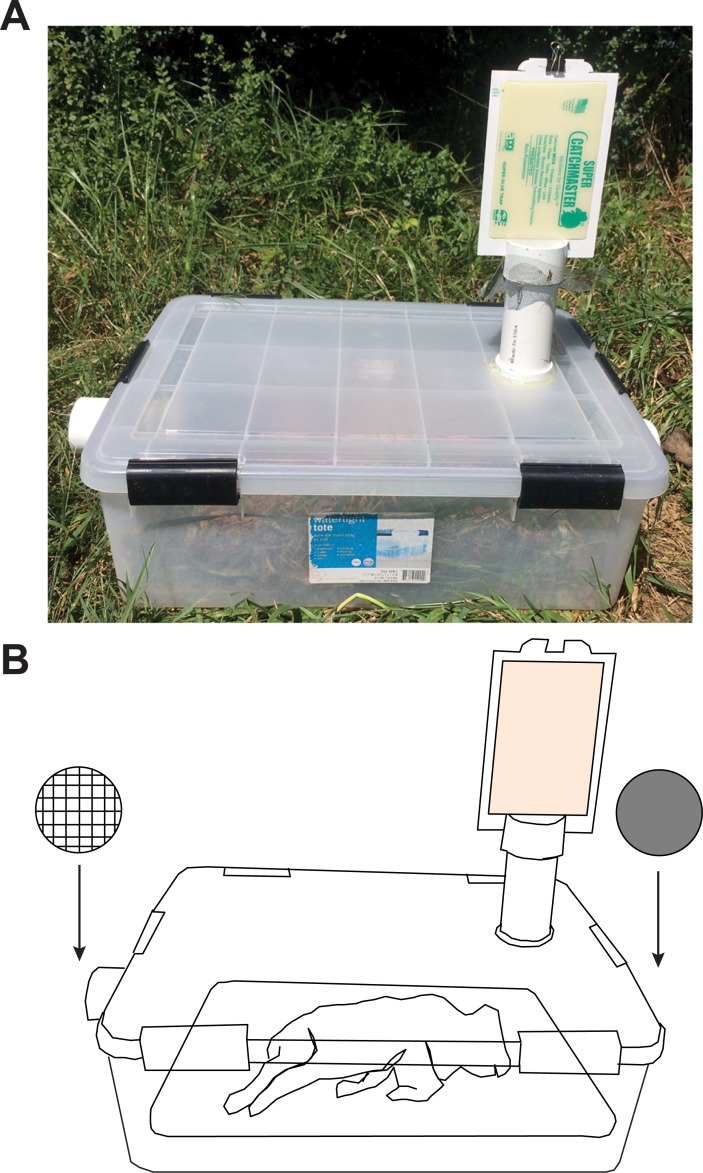
The vented-chamber passive sampling method. Photograph (**A**) and schematic (**B**) of the trap. The collection unit consisted of a 39 liters airtight chamber with PVC ports. Ports on the left and top of the box, with the orientation as shown, were kept open with mesh window screening. Thus, air could flow freely through the chamber, but insects could not enter it. The right port was capped. The top port, or chimney, opened to back-to-back unscented glue traps. Pig orientation in reference to the ports was always as shown in **B**.

Pigs were simultaneously sampled three times each day between noon and 18:00 hrs, beginning 24 hrs after their placement in the field (defined as day 1). Insect sampling was achieved through the passive, vented-chamber method, which directed thermally convected decomposition odors to a pair of sticky traps, as described in Cruise [14] (Fig 2). At each sampling event, each pig on a cafeteria tray was placed in the chamber and an airtight lid placed on top. Back-to-back unscented glue traps (Super Catchmaster, AP&G, Bayonne, New Jersey) were attached to the chimney atop the chamber and allowed to collect insects for 15 min. Each pig was sampled three times daily for seven consecutive days, with a 15 min rest period between sampling intervals. During the rest period, the pig and tray were removed from the chamber and placed on the ground, allowing decomposition and colonization to occur freely.

To sample beetles, hand-collections were performed on the pig both before and after its placement in the vented-chamber. Beetles were stored in 70% ethanol.

### Identifications

Orders Diptera and Coleoptera were the main sampling targets, with emphasis on necrophilous insects commonly used in forensic entomology for PMI determinations (Table 1) [15]. The local fauna of interest was also reported in Cammack et al. [12]. Because of their significant role as primary colonizers of decomposing bodies, calliphorid blow flies were further identified to species using Whitworth’s taxonomic key [17]. Beetles were identified with assistance from taxonomists in the North Carolina Plant Disease and Insect Clinic, as well as Almeida and Mise’s forensic Coleoptera key [18]. Insects on sticky traps were identified in situ.

**Table 1:** Forensically relevant insects identified across all samples and their ecological associations.

Order	Family	Genus and species ^a^	Ecological Association ^b^: Carrion (C), Insects (I), Dung (D)
**Diptera**	Calliphoridae	*Lucilia illustris* (Meigen)	C [15, 19, 20]
		*Lucilia coeruleiviridis* (Macquart)	C [15, 20]
		*Lucilia sericata* (Meigen)	C [15, 19]
		*Lucilia cuprina* (Wiedemann)	C [15]
		*Phormia regina* (Meigen)	C [15]
		*Cochliomyia macellaria* (Fabricius)	C [15, 20]
	Sarcophagidae	unknown	C [21]
	Muscidae	*Musca domestica* (Linnaeus)	D [15]
**Coleoptera**	Silphidae	*Necrophila americana* (Linnaeus)	C, I (dipteran predator and cannibal) [15]
	Histeridae	unknown	C, I, D (dipteran predator and cannibal) [15, 22]
	Staphylinidae	*Creophilus maxillosus* (Linnaeus)	C, I (dipteran predator) [15]
		*Platydracus* spp.	C, I (suspected dipteran predator) [15]
		unknown	C, I (dipteran predator) [5, 23, 24]
	Dermestidae	*Dermestes* spp.	C, I (cannibalistic) [15, 25]
	Scarabaeidae	*Onthophagus hecate* (Panzer)	D [15, 26]
		*Onthophagus pennsylvanicus* (Harold)	D [15, 26]
		*Onthophagus taurus* (Schreber)	D [15, 26]
		*Phanaeus vindex* (Macleay)	D [25, 26]

^a^ If no species are listed, taxonomic identifications ended at the family or genus level.

^b^ Insects that directly utilize carrion for feeding or breeding purposes are labeled “C,” predatory or cannibalistic insects are labeled “I,” and dung-feeding insects are labeled “D.”

### Ecological associations

Collected insects were further classified by their ecological associations. We defined these associations by three categories: carrion (C), predatory or cannibalistic insects (I), and dung (D) (Table 1). Carrion insects included those directly associated with carrion for feeding or breeding purposes, and they included all of the blow flies, flesh flies, and necrophagous beetles. Predatory or cannibalistic insects had ecological associations with another insect (hence “I”) in the carrion microhabitat. While many of the taxa opportunistically feed on dung, only insects with a preference for or well-documented association with dung, such as the scarab beetles, were classified as dung insects. Ecological associations were not mutually exclusive, with several insects fitting into more than one category.

### Statistical analysis

The number of taxa and the number of insects trapped on each of the eight pigs were compared on each day of decomposition with a one-way ANOVA and Tukey’s HSD in SAS 9.4 [27]. Linear discriminant analysis was conducted in JMP Pro 13.1.0 [28] on percentage representation of each taxon by day of decomposition.

## Results

### Decomposition pattern

**Fig 3:**
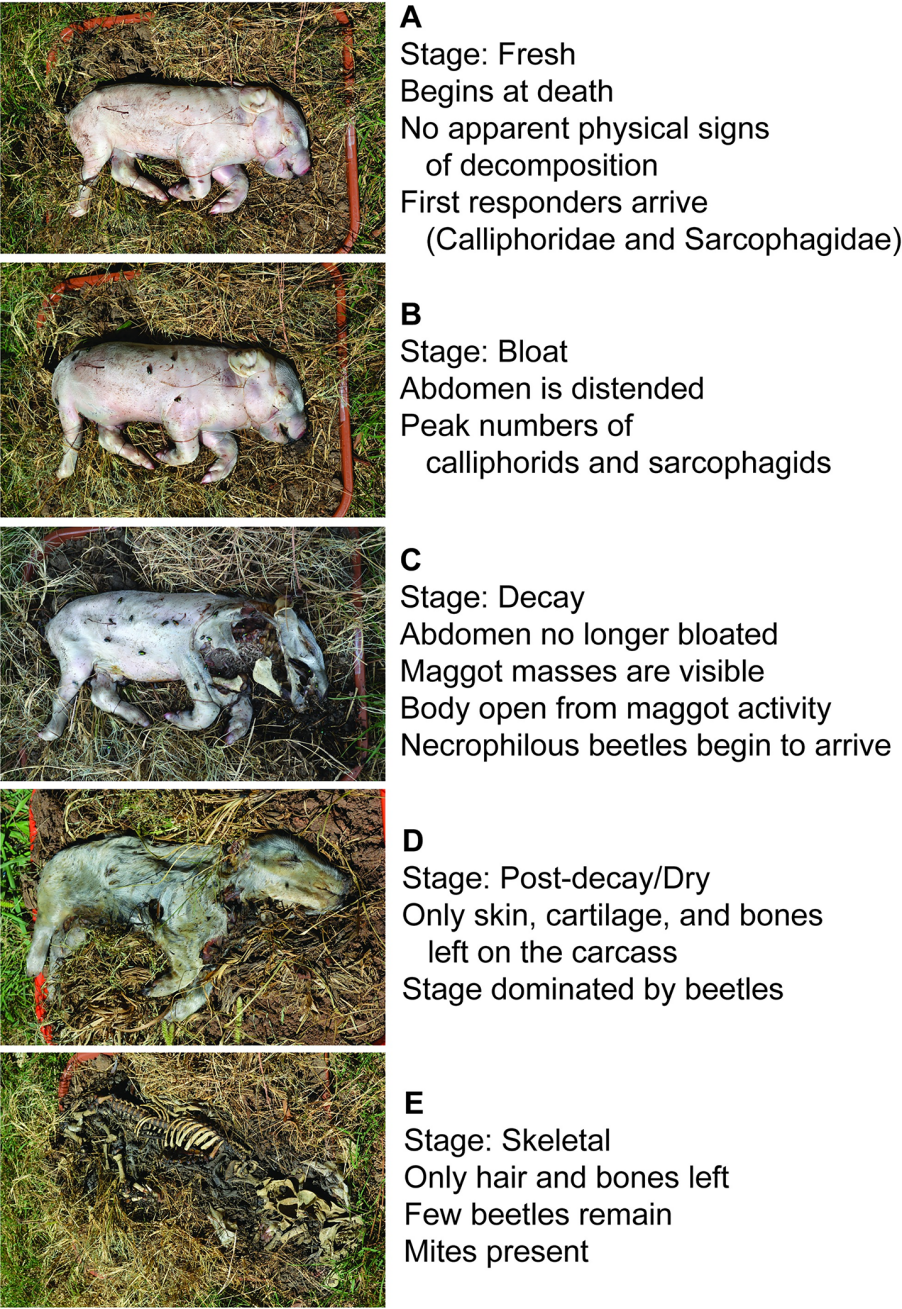
Decomposition stages of neonate pigs, showing pigs at each stage of decomposition and the corresponding characteristics of decomposition stages as defined by Kreitlow [16].

**Fig 4:**
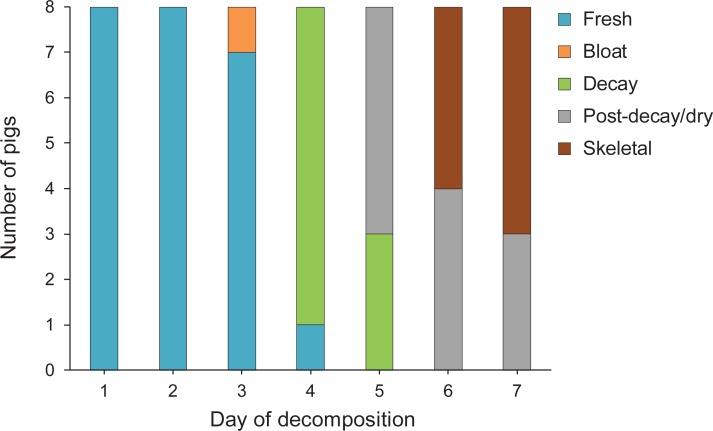
Number of pigs undergoing each of the five stages of decomposition throughout the 7-day experiment. All pigs (n = 8) were of the same weight (1.5 kg) and temperature (≤ 0°C) at the start of the experiment. Over half of the pigs were skeletonized by day 7 of the experiment.

Photographs and descriptions of decomposition stages of pigs are shown in Fig 3. Over half of the pigs (62.5%) fully progressed through decomposition (fresh to skeletal stages) over the 7 days of the experiment (Fig 4). Pigs were in the fresh stage for the longest duration, likely due to the cooler average temperatures during the first half of the experiment (Fig 1); rate of decomposition has been well correlated with ambient temperature [29, 30].

### Richness, overall abundance, and succession

**Fig 5:**
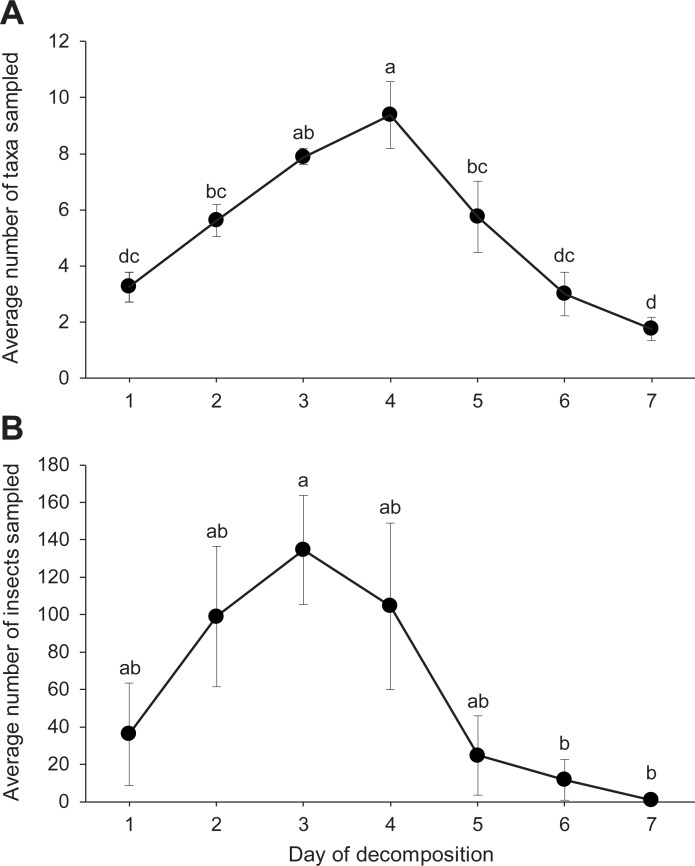
**Mean number of insect taxa (A) and insects (B) sampled from pigs at each day of decomposition.** Points and error bars show mean values ± SEM. Means labeled with the same letter are not significantly different (ANOVA, Tukey HSD, p < 0.05). (**A**) F = 11.77, df = 6, *p* < 0.0001; (**B**) F = 3.5, df = 6, *p* = 0.0059.

**Fig 6:**
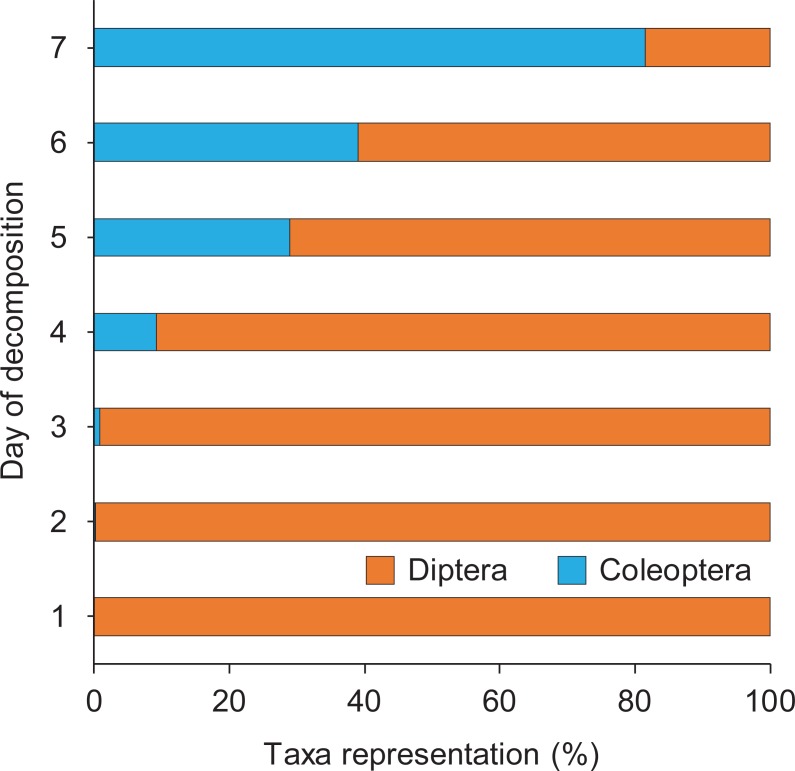
Percentage representation of dipteran and coleopteran taxa trapped or hand-collected from pigs on each day of the 7-day decomposition process.

In total, we trapped and hand-collected eight necrophilous fly taxa and 10 necrophilous beetle taxa, including four species of scarab beetles (Table 1). The average number of insect taxa varied by day but followed a general bell-shaped curve (Fig 5A), as did the number of necrophilous insects (Fig 5B). The relative abundance of dipterans and coleopterans varied with the decomposition stages of the pigs. Flies were most abundant, and they were most represented during the early stages (Fig 6). Necrophilous beetles of many species, however, arrived during and after the later decay stage, which started for most pigs on day 4 (Figs 4 and 6). By day 7, the number of insects trapped per pig averaged only 1.0 ± 0.76 insects (Fig 5B). At this time, 5 out of 8 (62.5%) pigs were in the skeletal stage of decomposition (Fig 4).

**Fig 7:**
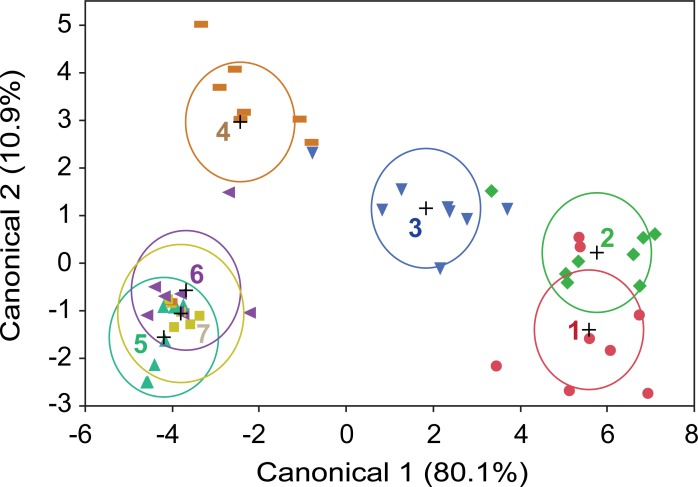
Linear discriminant analysis of 8 neonate pigs over 7 days of decomposition. The percentage representation of dipteran and coleopteran taxa (8 Diptera, 10 Coleoptera) trapped or hand-collected daily was used in the analysis. Color-coded numbers represent day of decomposition of each replicate pig. The plus (+) corresponds to each age group mean, and ellipses represent 95% confidence intervals for each mean.

Linear discriminant analysis of the relative abundance of each taxon by day showed that only the first two canonical relations had high eigenvalues and were significant (Canonical 1, *p* < 0.0001; Canonical 2, *p* = 0.0086). Linear discriminant analysis indicated significant differences among days of decomposition (Wilk’s lambda = 0.0024, *F* = 3.0866, *p* < 0.0001). Days 1 and 2, however, did not separate significantly, as indicated by overlap of their 95% confidence ellipses. Day 3 and day 4 separated clearly from each other and from all other days, whereas days 5–7 broadly overlapped (Fig 7). The rays in the biplot (not shown), representing the loading of taxa relative to the position of pig age groups, indicated that *Phormia regina*, *Lucilia sericata* and *Lucilia cuprina* associated (loaded) toward days 2–3, whereas *Cochliomyia macellaria* loaded towards day 4. The beetles generally loaded toward days 5–7.

### Diptera succession

**Fig 8:**
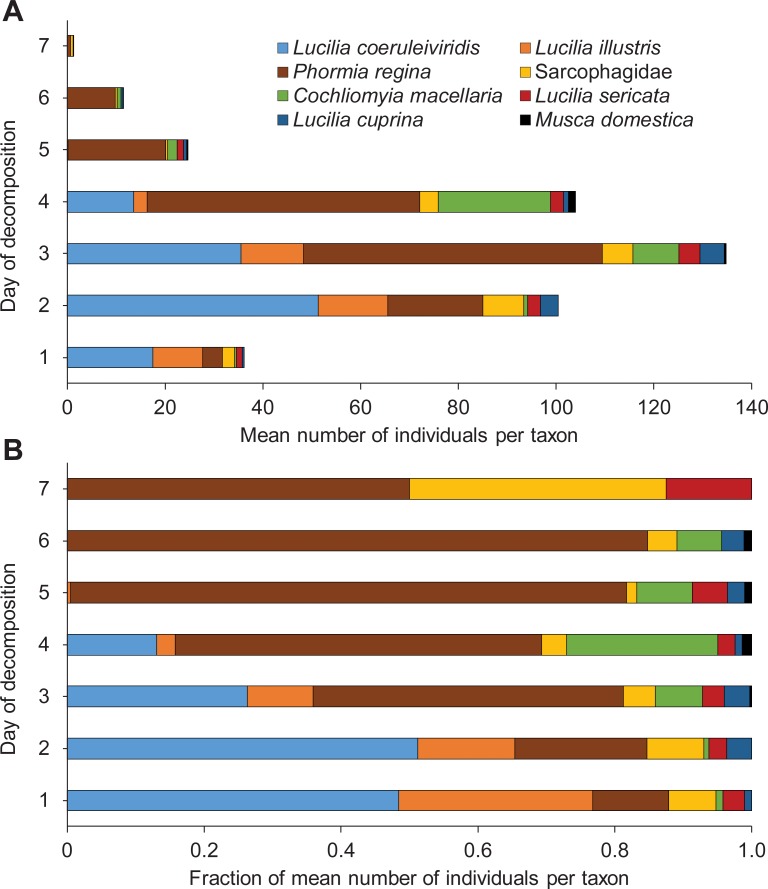
**Mean number (A) and percentage representation (B) of dipteran insects per taxon trapped or hand-collected daily from pigs during the 7-day decomposition process.** n = 8 pigs.

Table 2 details individual and total dipteran taxa abundance across all experimental days as well as the proportion of pigs each day from which each taxon was trapped on sticky traps. As expected, Diptera was the first major order of insects detected on the pig carcasses. In total, we collected 3,299 flies on all 8 pigs over 7 days. Blow flies and flesh flies were present on pigs as early as day 1 (Table 2). For the first three days of decomposition, flies were trapped from all 8 pigs, and the total dipteran abundance steadily increased between days 1 and 3 (Table 2, Fig 8).

**Table 2:** Total abundance of each dipteran taxon across all pigs by day.

Taxon	Day 1 ^a^	Day 2	Day 3	Day 4	Day 5	Day 6	Day 7	Taxon totals
***Lucilia illustris***	82 (0.875)	114 (1)	103 (1)	22 (0.375)	1 (0.125)	−	−	322 (1)
***Lucilia coeruleiviridis***	140 (0.75)	411 (1)	284 (0.875)	109 (0.75)	−	−	−	944 (1)
***Lucilia sericata***	9 (0.375)	21 (0.75)	34 (1)	21 (0.75)	10 (0.5)	−	1 (0.125)	96 (1)
***Lucilia cuprina***	3 (0.125)	29 (0.75)	40 (1)	8 (0.5)	5 (0.25)	3 (0.125)	−	88 (1)
***Phormia regina***	32 (0.25)	155 (0.75)	489 (1)	446 (0.875)	161 (0.5)	78 (0.25)	3 (0.125)	1364 (1)
***Cochliomyia macellaria***	3 (0.375)	6 (0.25)	75 (1)	184 (0.875)	16 (0.25)	6 (0.125)	−	290 (1)
**Sarcophagidae**	20 (0.5)	67 (0.875)	50 (0.875)	31 (0.75)	4 (0.375)	4 (0.25)	1 (0.125)	177 (1)
***Musca domestica***	−	−	3 (0.375)	12 (0.875)	2 (0.125)	1 (0.125)	−	18 (0.875)
**Total**	289 (1)	803 (1)	1078 (1)	833 (0.875)	199 (0.75)	92 (0.5)	5 (0.25)	3299 (1)

^**a**^ The proportion of pigs (out of eight) in which that taxon was trapped is indicated in parentheses. Boxes that are highlighted in gray indicate taxa trapped on all eight pigs (proportion: 8/8 = 1).

Dipteran abundance peaked on day 3, corresponding with the bloat stage for most pigs (Fig 8). Both the abundance and relative proportion of pigs with adult flies continually decreased after this (Table 2, Fig 8). Calliphorids were the most abundant group of flies found on the pigs (Table 2). All 6 calliphorid species were trapped on pigs beginning on day 1. These species were sampled from every pig on day 2 and/or day 3 (Table 2). Of the blow flies, *Lucilia coeruleiviridis* and *Lucilia illustris* were most abundant on days 1–2 and decreased in relative abundance over the next 4 days (Table 2, Fig 8). Conversely, *P*. *regina*, *C*. *macellaria*, *L*. *sericata* and *L*. *cuprina* were less represented on day 1 and increased in relative abundance from day 4 to day 7. The relative abundance of *P*. *regina* steadily increased from days 1 to 6, and it and *L*. *sericata* were the only calliphorid flies trapped on pigs on day 7 of decomposition, but in extremely low numbers.

Sarcophagids, while present on pigs during all seven days of decomposition (Table 2, Fig 8), never reached high overall abundance compared to calliphorids. Unlike the blow flies, sarcophagids were never sampled from all pigs on the same day. House flies, *M*. *domestica*, only appeared on pigs on days 3–6 (Fig 8) and were low in abundance throughout this entire interval (Table 2). Their abundance and proportion of pigs from which they were trapped peaked on day 4.

### Coleoptera succession

**Fig 9:**
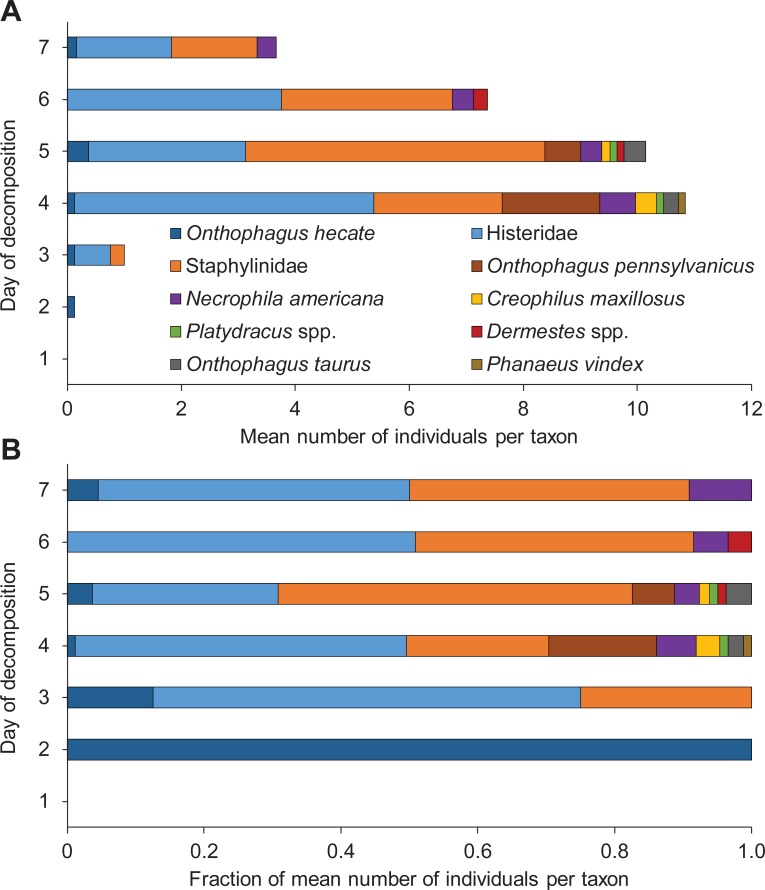
**Mean number (A) and percentage representation (B) of coleopteran insects per taxon trapped or hand-collected daily from pigs during the 7-day decomposition process.** n = 8 pigs.

Beetle activity began on day 2, a day later than the first fly arrival, and progressed through day 7 (Table 3, Figs 6 and 9). Beetle numbers on days 2 and 3 were low (< 10), but increased more than 10-fold between days 3 and 4. This coincided with a shift to the decay stage of pig decomposition (Fig 4). On all days except day 7, however, fly abundance was greater than beetle abundance (Fig 6).

**Table 3:** Total abundance of each coleopteran taxon across all pigs by day.

Taxon ^a^	Day 1	Day 2	Day 3	Day 4 ^c^	Day 5	Day 6	Day 7	Taxon totals
***Necrophila americana*** ^b^	−	−	−	5 (0.25)	3 (0.375)	3 (0.375)	2 (0.125)	13 (0.625)
**Histeridae** ^b^	−	−	5 (0.25)	42 (1)	22 (0.875)	30 (0.75)	10 (0.625)	109 (1)
***Creophilus maxillosus***	−	−	−	3 (0.375)	1 (0.125)	−	−	4 (0.5)
***Platydracus* spp.**	−	−	−	1 (0.125)	1 (0.125)	−	−	2 (0.25)
**Staphylinidae** ^b^	−	−	2 (0.25)	18 (0.875)	42 (1)	24 (0.875)	9 (0.5)	95 (1)
***Dermestes* spp.**	−	−	−	−	1 (0.125)	2 (0.125)	−	3 (0.25)
***Onthophagus hecate***	−	1 (0.125)	1 (0.125)	1 (0.125)	3 (0.25)	−	1 (0.125)	7 (0.5)
***Onthophagus pennsylvanicus***	−	−	−	12 (0.625)	5 (0.375)	−	−	17 (0.875)
***Onthophagus taurus***	−	−	−	2 (0.25)	3 (0.375)	−	−	5 (0.5)
***Phanaeus vindex***	−	−	−	1 (0.125)	−	−	−	1 (0.125)
**Daily totals**	−	1 (0.125)	8 (0.5)	85 (1)	81 (1)	59 (1)	22 (0.75)	256 (1)

^a^ All coleopteran taxa were hand-collected.

^b^ Taxa also trapped on the sticky traps of the vented-chamber.

^c^ In parentheses is the proportion of pigs (out of eight) in which that taxon was trapped. Boxes that are highlighted in gray indicate taxa trapped on all eight pigs (proportion: 8/8 = 1).

The successional pattern of beetles progressed in accordance with their ecological roles. Beetles that were categorized as both carrion-feeders and dipteran predators, including the silphid beetle *Necrophila americana*, histerid beetles, and staphylinid taxa, arrived after maggots were present on the body but before the carcass tissue was depleted (Tables 1 and 3). The arrival of these taxa occurred on either day 3 (histerid beetles) or day 4 (all others), when the pigs were either bloated or in the decay stage of decomposition (Fig 4). During these stages, the beetles could feed freely on either maggots or pig tissue.

Three of the four scarab taxa (*Onthophagus pennsylvanicus*, *Onthophagus taurus*, *and Phanaeus vindex*) did not arrive until day 4 (Table 3, Fig 9). These three taxa were present on pigs for only one or two days, likely due to the depletion of their preferred fecal resource. The remaining scarab, *Onthophagus hecate*, was collected in low numbers on pigs during 5 of the 7 days of the experiment.

Dermestid beetles were the latest arriving beetles, with their first arrival to the body on day 5 (Table 3). Most pigs were in the post-decay/dry stage at this time (Fig 4). Dermestid beetles were found only on days 5 and 6, likely because only hair and bone was left on most pigs after this point (Fig 3). Very few dermestid beetles (< 5) were sampled overall (Table 3, Fig 9).

## Discussion

From the outset, it is important to stress that our passive trap sampled necrophilous insects as they arrived at the carcass, before they directly interacted with the carcass, hence their attraction to this trap was solely based on olfactory cues. Therefore, the ecological succession we describe excluded other sensory modalities (e.g., visual cues), on-carcass interactions (e.g., competition, mating), and close-range decisions by the insects (e.g., whether to oviposit), because the arriving insects could use only volatile olfactory cues emanating from the chimney of the vented-chamber [14]. All the same, olfactory attraction alone was sufficient to create a comprehensive representation of succession that we could relate to clear taxonomic and ecological succession and to the successive decomposition stages of the pigs. These conserved successional patterns highlight the critical importance of olfaction in ecological succession of necrophilous insects.

Not surprisingly, we found that ecological roles helped to explain the general arrival sequence and relative abundance of insect taxa over time. Insects that directly used the carrion’s tissues for feeding or reproduction (Calliphoridae, Sarcophagidae) arrived first, during the fresh stage of decomposition, as expected [2, 31]. Adults oviposit (Calliphoridae) or larviposit (Sarcophagidae) on the body, and their young feed directly on animal tissues throughout development [3]. Competition between blow flies and flesh flies drives their relative population sizes on a carcass; generally, sarcophagid populations are limited by calliphorid population size, and larviposition by sarcophagids ensures that their larvae get an early start on consuming tissue before becoming outnumbered by calliphorids [21].

Carrion-associated fly activity increased through the bloat stage, as did the buildup of decomposition odors [29, 32, 33]. We observed the largest numbers of blow flies and flesh flies, as well as the highest trapping consistency across pigs for these taxa during the days when pigs were bloated (Table 2, Fig 4), consistent with previous work that evaluated succession on fully exposed carcasses [3, 16]. Blow fly and flesh fly activity declined after this point, likely due to dwindling resources. After the bloat stage, the feeding activity of large maggot masses begins to open the animal’s body, exposing the internal organs; tissue is then rapidly reduced as the maggots feed and grow [3].

Pivotal to our trap design was the hypothesis that necrophilous insects could assess resource suitability based on olfactory cues. Female blow flies are known to make this assessment prior to oviposition [34], and it follows that the number of adult flies arriving to oviposit and larviposit would decline as existing larvae consume the animal’s tissues and thus reduce the amount available for subsequent cohorts. While several fly species were attracted and trapped in small numbers on days 6 and 7, these insects would likely not oviposit or larviposit at this late stage in decomposition. Archer and Elgar [35] observed that most flies sampled after the decay stage of decomposition were non-gravid females searching for a protein source.

As blow fly and flesh fly activity decreased after the bloat stage, the activity of dung-associated and predatory/cannibalistic insects increased. The decay stage is known to favor necrophilous beetles that prey on abundantly available fly larvae and use the exposed carrion feces as a resource [3]. It was reassuring to find that these beetles also responded to stage-specific community and decomposition odors. With internal organs exposed in the decay stage, carrion insects had easy access to feces previously enclosed within the intestines. *M*. *domestica*, which is preferentially attracted to feces over carrion itself, as well as all scarab beetles were trapped and hand-collected in their highest numbers during this time (Tables 2 and 3, Figs 8 and 9) [15].

Insect activity was scarce during the skeletal stage. This stage is characterized by little insect activity, with mites instead being the main organisms associated with the remaining bones and hair [3, 16, 24]. Mite activity is rarely used in PMI determinations, and research on the subject is sparse [5]. Because we sampled mainly with sticky traps off the carcass, mites were excluded from this succession study.

Hand-collections recovered several taxa that were not readily sampled with the vented-chamber trap. The scarab *O*. *hecate* was hand-collected on days 2–5 and then again on day 7 (Fig 9). This beetle, like other paracoprid species, is known to tunnel below its fecal resource, which may explain why it was sampled even after above-ground fecal matter from carrion was depleted [36, 37]. Dermestid beetles, though carrion-associated, were not hand-collected until day 5, coinciding with the post-decay/dry stage for most pigs (Fig 4). These beetles feed on dry tissue, which is present on carrion during this stage of decomposition [15, 16, 38]. Few dermestid beetles (< 5) were collected overall, possibly because of their preference for hiding in small cavities within the hide [15].

Payne et al. [39] asserted that viewing decomposition stages as discrete events focuses more on physical changes in the carcass than the actual successional pattern of insects. While we agree with this view, we also found that the standard five-stage model of decomposition was useful in relating the ecology of the arriving insects to the physical dynamics of the pig. In many studies, stage boundaries are not synchronized with major faunal shifts [39]. We suspect that the rapid rate of decomposition in our study, likely due to carcass size and relatively warm, stable, precipitation-free environmental conditions and afternoon-only sampling, may have resulted in the appearance of a more synchronized timing of insect arrivals and more clearly defined stage boundaries. Maximum abundance of different taxa was better correlated with stage boundaries than was arrival time. Decomposition stage does not predict which individual taxa are present on carrion, but in our study, it explained the general pattern of arrival for insects fulfilling various ecological roles.

A significant motivation of this study was also a previous study [12] that was conducted at the same location. Although Cammack et al. [12] did not report quantitative results for each taxon, the black blow fly, *P*. *regina*, appeared to be the most commonly sampled species, as in our study (Table 2). Other flies that were trapped or hand-collected in both experiments included *C*. *macellaria*, *M*. *domestica*, and all four *Lucilia* species. *Chrysomya megacephala* and three *Calliphora* species were trapped by Cammack et al. [12] but not in our study. While Cammack et al. trapped *C*. *megacephala* in spring, summer, and fall, it was not trapped on sun-exposed carcasses. Also, none of the *Calliphora* species were collected in the summer [12], consistent with their cool weather preference and plausibly explaining their absence from our pigs which were placed in full sun in late summer.

Of the coleopteran taxa, *N*. *americana*, unidentified histerid beetles, *Creophilus maxillosus*, other unidentified staphylinids, and *Dermestes* species were found in both studies. We also documented several dung beetle species (*Onthophagus* and *Phanaeus*), but it is unknown whether these scarabs were sampled by Cammack et al. [12].

Several factors may explain the observed differences between the two studies: pig size, sampling time, sampling method, insects sampled (all stages vs. adults), and environmental conditions (level of exposure, landscape). Cammack et al. [12] used 10.2 kg juvenile pigs, several kilograms larger than our neonate (1.5 kg) pig models. Although larger pigs generally attract more flies than smaller pigs, and their decomposition rate may be slower, carcass size generally has little effect on the pattern of insect succession, as long as the same type of animal model is used and the difference in mass between the carcasses is relatively small (< 25 kg) [40–42]. When large and small carrion differ by > 25 kg, the succession of late colonizing insects from families Cleridae and Nitdulidae may differ, with these families being underrepresented on smaller carcasses [42]. The general similarity of our findings to those of Cammack et al. [12], and the similarity of both studies to previous investigations with human, swine, canine, and rat remains indicate that the size of the pig carcasses likely did not contribute substantially to the slight differences between our study and Cammack et al. [12].

Sampling time also differed between the two studies. Cammack et al. [12] sampled 2 hrs after morning civil twilight and 2 hrs before evening civil twilight, whereas we sampled between noon and 18:00 (18:00 is within the two hours before evening civil twilight). Interestingly, *C*. *megacephala*, collected by Cammack et al. but not in our study, is one of few blow flies known to oviposit at night [43]. Since Cammack et al. did not distinguish between larval and adult samples, it is possible that *C*. *megacephala* represented larvae that had developed from eggs oviposited overnight. Moreover, *C*. *megacephala* was not found on carcasses in a sun-exposed open field location [12] similar to our placement of the pigs.

We suspect that different sampling methods and environmental conditions contributed most to the successional differences in the same location. We coupled the vented-chamber approach, primarily to trap flies, with hand-collections of beetles, whereas Cammack et al. [12] used a modified vacuum and sweep net for adults and hand-collections for larvae and pupae. While a modified vacuum (different from Cammack et al.’s) was significantly more effective than sweep net during peak decomposition [13], comparisons across separate studies suggest that the vented-chamber was more effective than either of these approaches [14]. We emphasize again two important points. First, the vented-chamber method samples only newly arriving adults and not adults that are resting on the carcass or emerging from the carcass. And second, although *C*. *megacephala* was found to be an indicator species for the summer season in this location, it was not found in exposed sunny locations [12], and we did not find it in any of our sampling. The level of exposure (i.e., sun exposure, amount of tree cover) is known to affect faunal differences [1, 44].

Comparisons across successional studies depend on the availability of empirical sampling results of forensically relevant arthropods. Often however, data are reduced to community similarity or dissimilarity indices and statistically derived indicator species. While these metrics are obviously useful, data on relative abundance of taxa, peculiarities of common vs. rare species, and the temporal changes in community organization tend to be obscured in the analysis. For example, Cammack et al. [12] reported *C*. *macellaria* and *P*. *regina* as summer indicator species on sun-exposed pig carcasses. Our study in the same location confirmed that these two species respectively represented 8.8% and 41.3% of all the flies. *L*. *coeruleiviridis* (28.6%) and *L*. *illustris* (9.8%), a known summer active species, were also highly represented in our mid-September samples, possibly because of the summer-fall transition. A qualitative comparison of the two studies cannot be made, however, as the relative numbers of sampled adults and larvae were not reported by Cammack et al. [12].

Our quantitative analysis of succession on neonate pigs should provide a resource for local forensic entomology investigations, as well as a framework for an experimental approach for documenting ecological succession on carrion. Several aspects of this work are noteworthy: First, the use of neonate pigs, which are readily available, facilitated not only replication of this work, but also the ease of moving and manipulating carcasses. Secondly, standardization with the vented-chamber as a passive sampling method removed human bias and the inherent disturbance caused by active sampling with a sweep net or vacuum device. Finally, the high trapping efficiency of the vented-chamber makes it a useful model toward standardizing sampling so that successional patterns can be compared across geographic regions, seasons, and carrion types (see Results in [39, 45]). As discussed previously [14], this approach could be modified to increase the probability of trapping beetles which tend to orient to the carcass on the ground.

## Supporting information

S1 Dataset Supporting data. (XLSX)
